# Proteomic analysis reveals sex-specific biomarker signature in postural orthostatic tachycardia syndrome

**DOI:** 10.1186/s12872-020-01465-6

**Published:** 2020-04-22

**Authors:** Jasmina Medic Spahic, Fabrizio Ricci, Nay Aung, Erik Hallengren, Jonas Axelsson, Viktor Hamrefors, Olle Melander, Richard Sutton, Artur Fedorowski

**Affiliations:** 1 Department of Clinical Sciences, Malmö, Faculty of Medicine, Lund University, Clinical Research Center, 214 28 Malmö, Sweden; 2 Department of Internal Medicine, Skåne University Hospital, 214 28 Malmö, Sweden; 3 Department of Neuroscience, Imaging and Clinical Sciences, “G. d’Annunzio” University, 66100 Chieti, Italy; 4 Casa di Cura Villa Serena, Città Sant’Angelo, 65013 Pescara, Italy; 5 William Harvey Research Institute, NIHR Cardiovascular Biomedical Research Unit at Barts, Queen Mary University of London, London, UK; 6 Department of Clinical Immunology and Transfusion Medicine, Karolinska University Hospital, Stockholm, Sweden; 7 National Heart and Lung Institute, Imperial College, Hammersmith Hospital Campus, Ducane Road, W12 0NN, London, UK; 8 Department of Cardiology, Skåne University Hospital, Carl-Bertil Laurells gata 9, 214 28 Malmö, Sweden

**Keywords:** Postural orthostatic tachycardia syndrome, Autonomic nervous system diseases, Syncope, Biomarker, Cardiovascular disease

## Abstract

**Background:**

Postural orthostatic tachycardia syndrome (POTS) is a variant of cardiovascular (CV) autonomic disorder of unknown etiology characterized by an excessive heart rate increase on standing and orthostatic intolerance. In this study we sought to identify novel CV biomarkers potentially implicated in POTS pathophysiology.

**Methods:**

We conducted a nested case-control study within the Syncope Study of Unselected Population in Malmö (SYSTEMA) cohort including 396 patients (age range, 15–50 years) with either POTS (*n* = 113) or normal hemodynamic response during passive head-up-tilt test (*n* = 283). We used a targeted approach to explore changes in cardiovascular proteomics associated with POTS through a sequential two-stage process including supervised principal component analysis and univariate ANOVA with Bonferroni correction.

**Results:**

POTS patients were younger (26 vs. 31 years; *p* < 0.001) and had lower BMI than controls. The discovery algorithm identified growth hormone (GH) and myoglobin (MB) as the most specific biomarker fingerprint for POTS. Plasma level of GH was higher (9.37 vs 8.37 of normalised protein expression units (NPX); *p* = 0.002), whereas MB was lower (4.86 vs 5.14 NPX; *p* = 0.002) in POTS compared with controls. In multivariate regression analysis, adjusted for age and BMI, and stratified by sex, lower MB level in men and higher GH level in women remained independently associated with POTS.

**Conclusions:**

Cardiovascular proteomics analysis revealed sex-specific biomarker signature in POTS featured by higher plasma level of GH in women and lower plasma level of MB in men. These findings point to sex-specific immune-neuroendocrine dysregulation and deconditioning as potentially key pathophysiological traits underlying POTS.

## Background

Postural orthostatic tachycardia syndrome (POTS) is an autonomic disorder characterized by orthostatic intolerance and high prevalence among young women [1]. As etiology of POTS is largely unknown, effective therapeutic intervention for this syndrome has yet to be developed. Beyond genetic norepinephrine transporter deficiency, several theories have been proposed for the syndrome’s etiology, including antiadrenergic autoimmunity [2–4], baroreflex dysfunction [5], deconditioning [6], abnormal mast cell activation [1], excessive sympathetic drive and/or sympathetic denervation [7].

Targeted proteomics, yielding broad screening of circulating proteins with presumed role in cardiovascular pathology, offer promise as a tool for biomarker discovery. High-throughput multiplex protein arrays that rely on common methods such as polymerase chain reactions, require small sample volumes and are available at a fraction of the cost of large-scale platforms. Such a solution may provide an effective resource to discover disease pathways and identify novel therapeutic targets for individualised treatment based on biomarker profiling [8]. The proximity extension assay has been shown to be useful for biomarker discovery in cardiometabolic disease [9], immunology [10], cardio-oncology [11] and neuroscience research [12]. Moreover, multiprotein assays have been used to discover key mechanisms by which CV autonomic dysfunction is associated with increased CV morbidity and mortality [13, 14].

By applying multiple-protein screening based on proximity extension assay technology, we aimed to discover CV disease biomarkers associated with POTS in order to understand better the pathophysiology underlying this unexplained condition.

## Methods

### Study population

**Fig. 1:**
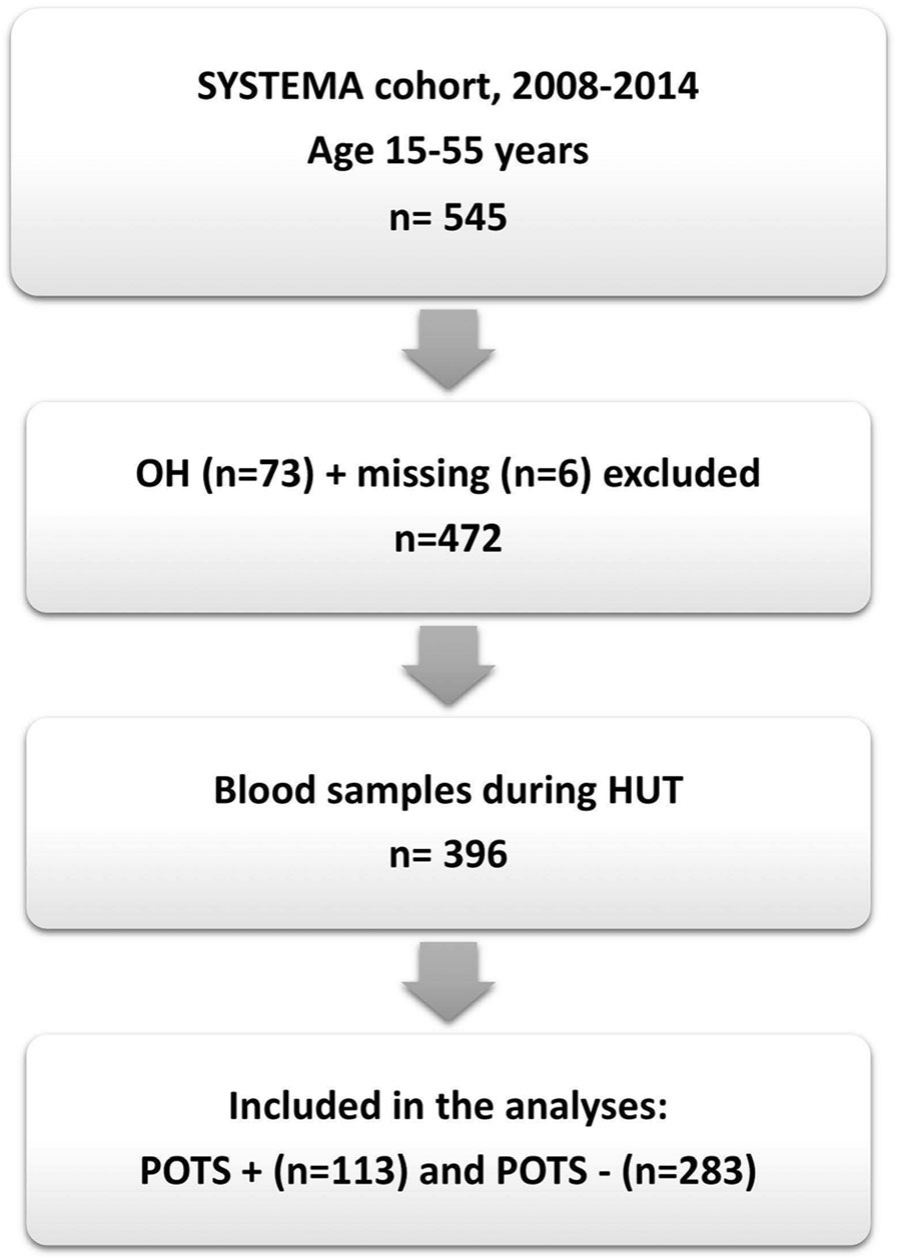
Flowchart of patient selection. HUT, headup tilt; OH, orthostatic hypotension; POTS, postural orthostatic tachycardia syndrome; SYSTEMA, Syncope Study of Unselected Population in Malmö

We analysed 994 consecutive patients from the Syncope Study of Unselected Population in Malmö (SYSTEMA). All patients were referred to our specialized syncope unit at Skåne University Hospital in Malmö due to unexplained syncope and/or symptoms of chronic orthostatic intolerance, and have been investigated by CV autonomic tests including head-up tilt testing (HUT), according to existing European guidelines [15]. From the cohort of 994 patients, we selected those age 15–50 years with available proteomics data and either diagnosis of POTS or normal hemodynamic response during passive head-up tilt test (Fig. 1).

The age filter was selected on previous epidemiological studies on POTS incidence [16, 17].As the study cohort (SYSTEMA) is basically a patient cohort and enrollment in the study demands an obligatory examination by tilt testing, normal asymptomatic controls were not available. Thus, in this situation, those, who were tilt-negative i.e. without hemodynamic changes corresponding to overt autonomic dysfunction including vasovagal syncope, orthostatic hypotension (OH) or POTS, were considered normal on the day tests were performed and were taken as ‘controls’.

The study protocol conformed to the ethical guidelines of the 1975 Declaration of Helsinki and was approved by The Regional Ethical Review Board of Lund University (No 82/2008).

All patients provided their written informed consent.

### Examination protocol

Patients were on their regular medication, except for CV drugs, which were discontinued at least 48 h prior to examination. Patients were told to fast for 2 h prior to examination but were allowed to drink water at will. Previous medical history was explored using a standard study questionnaire. During examination performed in the morning, the patients were placed on a tilt table and after a rest period for at least 10 min blood samples were collected through a venous cannula inserted in the forearm. As soon as the hemodynamic parameters were stable, a standard 70°HUT was carried out according to the Italian protocol recommended by European Society of Cardiology [18]. Beat-to-beat blood pressure and ECG were monitored by a validated non-invasive photoplethysmographic method (Nexfin monitor; BMEYE, Amsterdam, the Netherlands) with a wrist unit and finger cuff of appropriate size [19]. POTS was defined as the reproduction of symptoms of orthostatic intolerance associated with heart rate increase > 30 bpm; or sinus tachycardia > 120 bpm during first 10 min of HUT; or increase > 40 beats/min for those < 18 years of age, with history of orthostatic intolerance for at least 6 months [20]. Patients with signs of OH i.e. with systolic blood pressure (BP) drop ≥20 mmHg or diastolic BP drop ≥10 mmHg [21] during tilt testing were excluded.

### Proteomics analysis

Plasma biomarkers were measured from blood samples (total volume: 30 ml) that had been first centrifuged, then stored as 16 × 250 μL aliquots of EDTA plasma in plastic thermotubes, and frozen at − 80 °C. Samples were thawed and examined by the Proximity Extension Assay technology enabling high-throughput, multiplex immunoassays that measure 92 CVD-related human proteins across 96 samples simultaneously using only one microliter of plasma.

Concisely, 92 pairs of oligonucleotide-labeled antibodies (probes) were used to detect the corresponding target proteins in the plasma sample. When the two antibodies are brought in proximity, a new polymerase-chain reaction (PCR) target sequence is formed. The complex is subsequently detected and quantified by standard real-time PCR. Quantitative PCR quantification cycles corrected for technical variation by the Inter-plate Control generate Normalized Protein Expression (NPX) values, which are arbitrary units on log2 scale. A higher NPX value corresponds to a higher protein level. More information about PEA technology, assay performance, quality control and validation is available at the Olink webpage (http://www.olink.com).

### Statistical analysis

Patients with available proteomics dataset and a definitive diagnosis of POTS (*n* = 113) or normal hemodynamic response to HUT (*n* = 283), i.e. without vasovagal syncope, OH and abnormal postural tachycardia, and importantly without prevalent cardiovascular disease or hypertension, were selected. Missing data was imputed with multiple imputation by chained equations (MICE) approach. We used predictive mean matching for continuous variables, logistic regression for binary variables, and polytomous regression for categorical variables. All covariates were included in the imputation models. The maximum iteration was set at 20 and convergence was confirmed by visual examination of trace plots (Online Fig. S1).

We explored the change in cardiovascular proteomics associated with POTS through a sequential two-stage process including supervised principal component analysis and univariate ANOVA with Bonferroni correction, as previously described [13, 14, 22].

Univariate and multivariate ordinary least square linear regression and logistic regression models were conducted for bivariate correlation between plasma level of biomarkers and maximum orthostatic heart rate change (ΔHR) or POTS status, respectively. Multivariate regression models were adjusted for age, sex and body-mass index (BMI). We also performed sensitivity analyses stratified by sex and quantile-regression analysis in order to identify differing relationships at different quartiles of HR changes during HUT. The mean estimates and standard errors of the beta-coefficients for the imputed datasets were combined under Rubin’s rules [23, 24]. Statistical analysis was performed using IBM SPSS Statistics version 25 (SPSS Inc., Chicago, IL, USA) and R Statistical Software (version 3.4.4; R Foundation for Statistical Computing, Vienna, Austria).

## Results

We analyzed 396 patients (female, 69%; age range, 15–50 years) including 113 POTS and 283 controls. Basic characteristics of the study population stratified by POTS status are shown in Table 1. Biomarkers with > 35% missing values, i.e. leptin, were excluded (see Online Table S1), inasmuch pairwise complete data are required for running principal component analysis.

**Table 1:** Baseline characteristics of the study population

Characteristic	POTS- (*n* = 283)	POTS+ (*n* = 113)	*P*-value
**Age (years)**	31.5 (9.8)	26.3 (8.4)	< 0.001
**Female sex, n (%)**	189 (66.8)	83 (73.5)	0.241
**BMI, Kg/m**^**2**^	24.3 (4.1)	22.7 (3.5)	< 0.001
**SBP supine, mmHg**	119.9 (14.2)	120.4 (14.2)	0.833
**DBP supine, mmHg**	69.9 (8.2)	70.2 (8.2)	0.788
**HR supine, bpm**	68.7 (11.9)	71.1 (11.6)	0.084
**SBP HUT min, mmHg**	112.2 (13.4)	107.6 (16.2)	0.003
**DBP HUT min, mmHg**	71.8 (9.1)	72.5 (10.6)	0.552
**HR HUT max, bpm**	84.7 (13.8)	112.4 (15.6)	< 0.001
**Smoking, n (%)**	58 (20.5)	16 (14.2)	0.188

*P*-values for differences between the groups are shown as mean and SD for continuous variables and as percentages for categorical variables.; *DBP* Diastolic blood pressure; *HR* Heart rate; *HUT* min/max, lowest/highest value during passive head-up tilt test; *IHD* Ischemic heart disease; *POTS* Postural orthostatic tachycardia syndrome; *SBP* Systolic blood pressure

At the stage of biomarker signature discovery, univariate logistic regression was performed for each biomarker and regression coefficients were standardised by dividing the coefficient with its standard error. All possible thresholds (standardised coefficient (θ) ranging from minimum to maximum with 0.05 increments) were used to select groups of biomarkers and build principal components (PCs). POTS status was then regressed onto the first two PCs from each group of biomarkers using the binomial link function. The threshold providing the best classification accuracy (POTS+ vs controls) was selected by ten-fold cross-validation and the following 23 biomarkers were identified: TIM, PSGL1, CTSL1, MB, VEGFD, PIGF, MMP1, GDF15, FAS, TF, AM, UPAR, TNFR2, TRAIL, MCP1, TRAILR2, OPG, CASP 8, HGF, CD40L, GH, PTX3 (see Online Table S2 for acronyms).

**Fig. 2:**
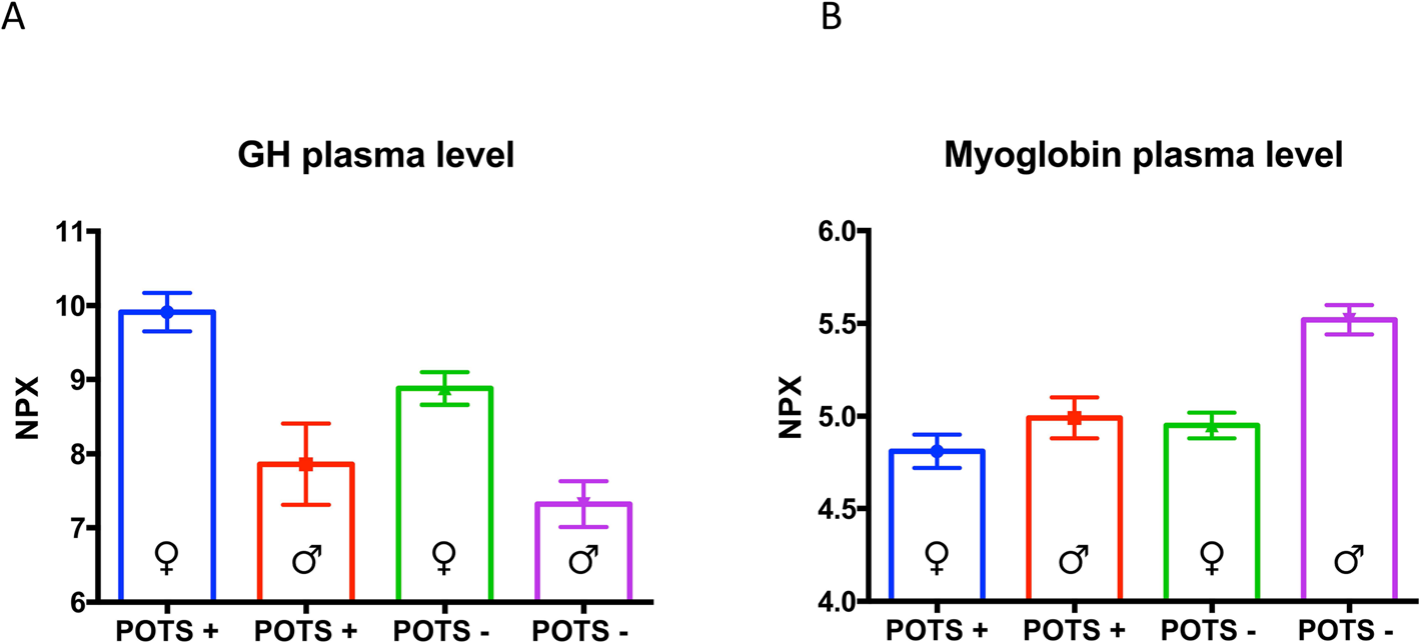
The plasma levels of growth hormone (GH) (panel A) and myoglobin (panel B), expressed on Normalised Protein Expression (NPX) on a log2 scale, are presented in relation to POTS and sex status. Data are shown as a box and whisker plot with median in the box and the whiskers representing the 5th and 95th percentiles in relation to plasmatic biomarker level

At the stage of biomarker verification analysis, nine PCA-selected proteins differed significantly in pairwise comparison, but only GH and MB attained significance after Bonferroni correction (Table 2). Plasma levels of GH were significantly higher in POTS women compared with POTS men (*p* = 0.0002), and both male (*p* < 0.0001) and female controls (*p* = 0.003) (Fig. 2). Conversely, plasma level of MB were significantly lower in POTS men compared with male controls (*p* = 0.0009).

**Table 2:** High throughput multiplex analysis of biomarkers selected by supervised multivariate principal component analysis. Plasma concentrations of the assessed proteins are expressed on a log2-scale. Inter-group differences were assessed using analysis of variance method. *Bonferroni-corrected significant values (*p* < 0.0022)

Biomarker	POTS+ (***n*** = 113)	POTS- (***n*** = 283)	***P***-value*
**TIM**	4.32 (0.08)	4.49 (0.05)	0.078
**PSGL1**	0.69 (0.04)	0.8 (0.02)	0.009
**CTSL1**	5.3 (0.04)	5.46 (0.03)	0.004
**MB**	4.86 (0.07)	5.14 (0.06)	0.0020*
**VEGFD**	6.7 (0.06)	6.82 (0.03)	0.062
**PlGF**	7.1 (0.05)	7.21 (0.04)	0.131
**MMP1**	3.04 (0.14)	3.41 (0.09)	0.029
**GDF15**	8.22 (0.07)	8.42 (0.05)	0.058
**FAS**	6.93 (0.04)	7.02 (0.03)	0.045
**TF**	5.53 (0.04)	5.61 (0.03)	0.120
**AM**	5.83 (0.09)	6.05 (0.05)	0.029
**UPAR**	9.66 (0.03)	9.72 (0.03)	0.127
**TNFR2**	4.82 (0.04)	4.9 (0.03)	0.116
**TRAIL**	8.44 (0.04)	8.52 (0.03)	0.183
**MCP1**	3.07 (0.05)	3.24 (0.05)	0.014
**TRAILR2**	1.21 (0.03)	1.28 (0.02)	0.080
**OPG**	9.16 (0.04)	9.25 (0.03)	0.095
**CASP8**	1.45 (0.07)	1.33 (0.05)	0.126
**HGF**	6.22 (0.04)	6.34 (0.04)	0.030
**CD40L**	8.57 (0.11)	8.6 (0.07)	0.647
**GH**	9.37 (0.26)	8.37 (0.2)	0.0019*
**PTX3**	1.38 (0.06)	1.28 (0.04)	0.124
**SRC**	7.66 (0.07)	7.77 (0.03)	0.087

In multivariate regression analysis adjusted for age (Fig. S1) and BMI and stratified by sex both POTS status and maximum orthostatic ΔHR were significantly associated with lower MB level in men and higher GH level in women (Tables 3, 4, and Online S3, S4).

**Table 3:** Sex-specific relationship between POTS status and selected biomarkers in univariate and multivariate regression analysis

Biomarker	Women					
	Univariate			Multivariate^a^		
	***OR***	95% CI	***P***-value	***OR***	95% CI	***P***-value
**MB**	0.92	0.81–1.04	0.193	0.94	0.83–1.07	0.378
**GH**	1.04	1.01–1.06	0.003	1.03	1.00–1.06	0.022
	Men					
**MB**	0.77	0.67–0.89	0.001	0.80	0.70–0.93	0.004
**GH**	1.01	0.98–1.04	0.422	1.00	0.97–1.03	0.981

^a^Adjusted for age and body mass index

**Table 4:** Sex-specific relationship between changes in heart rate during head-up tilt test and selected biomarker in univariate and multivariate regression analysis

Biomarker	Women					
	Univariate			Multivariate^a^		
	***β***	95% CI	***P***-value	***β***	95% CI	***P***-value
**MB**	−1.81	−5.35 to 1.72	0.317	−0.66	−4.35 to 3.03	0.726
**GH**	1.28	0.48 to 2.08	0.002	0.95	0.13 to 1.76	0.024
	**Men**					
	***β***	**95% CI**	**P-value**	***β***	**95% CI**	**-value**
**MB**	−7.44	−12.05 to −2.82	0.003	−5.0	−9.46 to − 0.54	0.03
**GH**	0.52	−0.53 to 1.58	0.331	0.06	−0.94 to 1.06	0.908

^a^Adjusted for age and body mass index

Quantile regression analyses investigating the relationships between selected biomarkers and quartiles of ΔHR did not reveal any obvious threshold effect or step function.

## Discussion

Using a novel high-throughput proteomics platform, we examined 92 cardiovascular plasma biomarkers in patients diagnosed with postural orthostatic tachycardia syndrome and controls with normal orthostatic response. We identified higher plasma levels of growth hormone and lower plasma levels of myoglobin in patients with POTS, whereas other biomarkers did not significantly differ between the two groups. We also documented sex-specific patterns of significance where lower MB level in men and higher GH level in women were independently associated with both binary POTS status and changes in heart rate during head-up tilt test, even after adjustment for age and BMI.

### Growth hormone

Higher levels of growth hormone in patients with postural orthostatic tachycardia syndrome, notably in females, was an unexpected finding that deserves detailed commentary. GH is an anabolic neuropeptide regulating carbohydrate and lipid metabolism via complex interactions with insulin and insulin-like growth factor-1 (IGF-1). The secretion of GH from the anterior pituitary gland is stimulated by growth hormone releasing hormone (GHRH) and inhibited by somatostatin and a negative feedback loop of IGF-1 [25]. The hormone is released in a pulsatile manner with significant circadian rhythm and peak discharge occurring at night-time, approximately one hour after sleep onset [26]. Previous studies reported higher incidence of CV events related to increased levels of GH [26]. Since long-term prognosis in POTS patients is still unknown, the effects of increased GH levels on CV outcome in this patient population remain to be explored.

There is a number of possible mechanistic explanations for higher levels of GH observed in POTS patients. Firstly, an increase in plasma GH concentration could be the result of proinflammatory cytokines acting as negative regulatory signals fine-tuning the action of hormones and growth factors. Tumour necrosis factor alpha and interleukin-1 beta are believed to cause IGF-1 resistance by weakening downstream signalling in myoblasts and this could eventually cause increased release of GH due to negative feedback [27]. This hypothesis could be tested in future studies comparing the levels of inflammatory mediators in POTS with healthy subjects. The current body of knowledge is very sparse and with only IL-6 reported to be elevated in POTS [28].

Secondly, it has been shown that POTS patients present with autoantibodies against alpha- and beta-receptors, which belong to the G-protein coupled receptor (GPCR) family of rhodopsin type [3]. Receptors for GHRH (GHRHr) are distributed on the anterior pituitary gland, which also belong to GPCR family, though of slightly different secretin type. Hypothetically, higher levels of GH might be the result of abnormal stimulation of GHRHr by circulating anti-GPCR antibodies. Nevertheless, the presence of such specific and functionally active anti-GHRHr autoantibodies has not been yet demonstrated in POTS patients.

Thirdly, patients with POTS usually have a lean body type with lower BMI which could be related to increased lipolysis due to higher GH [29]. Unfortunately, plasma insulin levels or HbA1c were not tested in this group of patients.

Fourthly, octreotide - a somatostatin analogue classically used to control hypersomatotropism in acromegaly through the inhibition of GH action and GH secretion [30] – has been reported to be an effective treatment for POTS patients by reducing upright tachycardia and symptoms of orthostatic intolerance due to its splanchnic vasoconstrictor effect [31]. Splanchnic blood flow has been shown indeed to be increased in the supine posture and to progressively increase during incremental tilt in POTS patients [32]. In the absence of peripheral sympathetic denervation, locally mediated vasodilation - involving vasoactive autacoids such as vasoactive intestinal polypeptide, substance P, calcitonin gene-related peptide, and nitric oxide - has been proposed as a possible explanation for splanchnic pooling. Interestingly, insulin-like growth factor-1 (IGF-1), that is synthesized in the liver, is secreted into the blood under the control of GH, and is known to induce peripheral vasodilation via NO synthase and/or potassium channel activity [33]. It may, therefore, provide a possible mechanistic link among the observed high levels of GH, splanchnic pooling and octreotide efficacy in reducing symptoms in POTS patients.

Finally, the relative syndrome-dependent inactivity among POTS patients might hypothetically lead to a reversal or gross disturbance in circadian patterns of GH release. However, deconditioning as an underlying pathophysiology of POTS and to possibly related syndromes such as chronic fatigue syndrome has not been supported by recent studies, and other mechanisms such as low ventricular filling have been proposed [34, 35]. It remains to be demonstrated if physical training may reverse GH level abnormality.

The influence of gender on serum concentrations of GH has been the object of previous investigations [36, 37]. In many species, including rats, mice, and humans, the temporal pattern of pituitary GH secretion is sex-specific (episodic in males, more frequent in females) and leads to sex differences in downstream signaling pathways in target tissues [38]. In our study blood samples were obtained in the morning after two-hour fasting, which ascertained stabilisation of GH concentration, but it has been also reported as a possible explanation for the observed high concentrations of GH in women, as if something in the morning - which may be regarded as very mild stress of fasting - could trigger a GH burst in almost all of the women but in very few of the men [37]. This could be because of gender differences in the sensitivity of the pituitary or hypothalamus to the GH-releasing effects of mild stress. Furthermore, there is quite robust evidence in the literature to suggest that endogenous estrogens play a major role in increased GH secretion in women compared with men [39]. Interestingly, in our study we observed POTS women presenting with significantly higher levels of GH than women without POTS. This could be the result of (i) complex interactions amongst sex-related and sex-unrelated immune-neuroendocrine mechanisms, (ii) sexually dimorphic patterns of GHRH secretion (iii) impaired cerebrovascular autoregulation [40], (iv) chronic deconditioning [41], and/or (v) hitherto unknown pathways.

### Myoglobin

Myoglobin, an oxygen and iron binding protein found in muscle cells, is usually increased when muscle tissue damage occurs though small amounts are normally present in plasma. Our analysis revealed decreased plasma myoglobin in POTS patients, notably males, compared with controls. The results are difficult to interpret as the method used for protein detection in this study provides only relative values within the analyzed sample, not absolute values that could be translated to clinically useful cut-off levels. Of note, the values reported here are likely not indicative of muscle damage, since patients were free of such clinical suspicion at inclusion. More likely, the levels detected here are linked to small amounts of myoglobin found in plasma in the absence of muscle damage. Lower myoglobin levels found in POTS might be a result of immobilisation and limited physical activity in those patients, possibly a consequence of cardiovascular deconditioning or chronic fatigue [1]. Thus, although deconditioning may not be causally related to POTS [34], such deconditioning may be the result of reduced exercise tolerance experienced by many POTS patients [42]. In addition, the lower myoglobin levels found in POTS may be seen to parallel reduced iron stores, which is, in turn, associated with POTS [43]. Unfortunately, data on iron status was not available in our study population. Moreover, considering the role of myoglobin in muscle metabolism [44], one may also hypothesize that myoglobin may, indeed, also have a role in the pathophysiology of POTS, even if such hypotheses are highly speculative at this stage.

Even though we did not measure lean body mass, POTS patients in our study did have lower BMI. It remains to be explored why this association is limited to men only.

Beyond growth hormone and myoglobin, we could observe aberrations in other proteomics biomarkers, although not achieving the adjusted significance level. However, the overall impression was that there were only slight differences in the analysed biomarkers. It may indicate that POTS is an inflammatory condition involving hitherto unknown pathophysiological mechanisms deserving further explorative and experimental studies.

### Final remarks

This study points the way toward application of other proteomics technology in POTS research. For instance, it might be useful to use similar technology to screen a large number of antibody variable region epitope sequences for coexisting antibodies that might be directed toward candidate G-protein coupled receptors considered relevant to POTS [3, 45] and related conditions such as chronic fatigue syndrome.

### Limitations

There are some limitations that must be addressed. Firstly, our control group included symptomatic individuals, who had normal hemodynamic response to tilt testing, but were referred to our center due to unexplained syncope and/or symptoms of orthostatic intolerance.

Secondly, this is a single-center experience with limited generalisability, also due to uneven sex distribution and lack of age matching; accordingly, and in the need of an external validation cohort, our findings have to be interpreted as hypothesis-generating.

Thirdly, our findings are based on one-off measurement precluding information about causality and temporal correlation of selected biomarkers with the progression of the disease, onset and burden of clinical symptoms.

Fourthly, we acknowledge the lack of information about menstrual cycle and use of hormonal contraceptives, as previous studies demonstrated that the hormonal fluctuations that occur during the normal menstrual cycle may alter autonomic regulation of blood pressure during various environmental stimuli [46], and the intake of exogenous estrogen has been shown to increase plasma levels of GH [37].

Fifthly, we recognize the lack of plasma GH determination on clinically validated high-sensitivity chemiluminescence sandwich immunoassay platforms for direct correlation with GH levels measured with PEA technology.

Finally, in order to rule-out false positive signals, our findings should be validated with alternative technologies enabling sensitive and robust detection and quantification of biomarkers. However, the use of a proximity extension assay technique, with the requirement for a dual binding event ensuring minimal noise signal, and the robust discovery algorithm would make a false positive result very unlikely.

## Conclusions

Our study confirms and extends the concept that high throughput multiplex analysis for protein profiling may considerably improve the understanding of POTS. Targeted cardiovascular biomarkers profiling based on proximity extension assay technology identified higher plasma level of growth hormone and lower plasma level of myoglobin respectively in women and men with POTS compared with subjects presenting normal hemodynamic response during head-up tilt test. This observation would be in keeping with the presence of distinct and sex-specific pathophysiological pathways underlying this unexplained syndrome.

## Supplementary information

**Additional file 1.**

**Additional file 2: Table S1.** Absolute and percentage missingness data. **Table S2.** Multiplex Cardiovascular Disease Panel: Biomarker List

**Additional file 3.**

**Additional file 4.**

## Data Availability

Supporting data will be made available upon request to the corresponding authors.

## Abbreviations

ANOVA: Analysis of variance
BMI: Body mass index
CV: Cardiovascular
GH: Growth hormone
GHRH: Growth hormone releasing hormone
GHRHr: Growth hormone releasing hormone receptors
GPCR: G-protein coupled receptor
HUT: Head-up tilt testing
IGF-1: Insulin-like growth factor-1
HR: Heart rate
MB: Myoglobin
NPX: Normalised protein expression units
PICO: Problem/patient/population intervention/indicator comparison outcome
POTS: Postural orthostatic tachycardia syndrome
SYSTEMA: Syncope study of unselected population in Malmö

## References

[1] Fedorowski A (2019). Postural orthostatic tachycardia syndrome: clinical presentation, aetiology and management. J Intern Med.

[2] Li H, Yu X, Liles C, Khan M, Vanderlinde-Wood M, Galloway A, Zillner C, Benbrook A, Reim S, Collier D (2014). Autoimmune basis for postural tachycardia syndrome. J Am Heart Assoc.

[3] Fedorowski A, Li H, Yu X, Koelsch KA, Harris VM, Liles C, Murphy TA, Quadri SMS, Scofield RH, Sutton R (2017). Antiadrenergic autoimmunity in postural tachycardia syndrome. Europace.

[4] Vernino S, Stiles LE (2018). Autoimmunity in postural orthostatic tachycardia syndrome: current understanding. Auton Neurosci.

[5] Muenter Swift N, Charkoudian N, Dotson RM, Suarez GA, Low PA (2005). Baroreflex control of muscle sympathetic nerve activity in postural orthostatic tachycardia syndrome. Am J Physiol Heart Circ Physiol.

[6] Fu Q, Vangundy TB, Galbreath MM, Shibata S, Jain M, Hastings JL, Bhella PS, Levine BD (2010). Cardiac origins of the postural orthostatic tachycardia syndrome. J Am Coll Cardiol.

[7] Jacob G, Costa F, Shannon JR, Robertson RM, Wathen M, Stein M, Biaggioni I, Ertl A, Black B, Robertson D (2000). The neuropathic postural tachycardia syndrome. N Engl J Med.

[8] Lind L, Arnlov J, Lindahl B, Siegbahn A, Sundstrom J, Ingelsson E (2015). Use of a proximity extension assay proteomics chip to discover new biomarkers for human atherosclerosis. Atherosclerosis.

[9] Nowak C, Carlsson AC, Ostgren CJ, Nystrom FH, Alam M, Feldreich T, Sundstrom J, Carrero JJ, Leppert J, Hedberg P (2018). Multiplex proteomics for prediction of major cardiovascular events in type 2 diabetes. Diabetologia.

[10] Abrahamsson A, Rzepecka A, Dabrosin C (2017). Equal pro-inflammatory profiles of CCLs, CXCLs, and matrix Metalloproteinases in the extracellular microenvironment in vivo in human dense breast tissue and breast Cancer. Front Immunol.

[11] Vohra A, Asnani A (2018). Biomarker discovery in cardio-oncology. Curr Cardiol Rep.

[12] Brann E, Fransson E, White RA, Papadopoulos FC, Edvinsson A, Kamali-Moghaddam M, Cunningham JL, Sundstrom-Poromaa I, Skalkidou A. Inflammatory markers in women with postpartum depressive symptoms. J Neurosci Res. 2018:1–13. 10.1002/jnr.24312. 10.1002/jnr.24312 30252150

[13] Johansson M, Ricci F, Aung N, Sutton R, Melander O, Fedorowski A. Inflammatory biomarker profiling in classical orthostatic hypotension: insights from the SYSTEMA cohort. Int J Cardiol. 2018;259:192–7. 10.1016/j.ijcard.2017.12.020 29579600

[14] Johansson M, Ricci F, Aung N, Sutton R, Melander O, Fedorowski A (2018). Proteomic profiling for cardiovascular biomarker discovery in orthostatic hypotension. Hypertension.

[15] Moya A, Sutton R, Ammirati F, Blanc JJ, Brignole M, Dahm JB, Deharo JC, Gajek J, Gjesdal K, Krahn A (2009). Guidelines for the diagnosis and management of syncope (version 2009): the task force for the diagnosis and management of Syncope of the European Society of Cardiology (ESC). Eur Heart J.

[16] Thieben MJ, Sandroni P, Sletten DM, Benrud-Larson LM, Fealey RD, Vernino S, Lennon VA, Shen WK, Low PA (2007). Postural orthostatic tachycardia syndrome: the Mayo clinic experience. Mayo Clin Proc.

[17] Hamrefors V, Spahic JM, Nilsson D, Senneby M, Sutton R, Melander O, Fedorowski A (2017). Syndromes of orthostatic intolerance and syncope in young adults. Open Heart.

[18] Bartoletti A, Alboni P, Ammirati F, Brignole M, Del Rosso A, Foglia Manzillo G, Menozzi C, Raviele A, Sutton R (2000). 'The Italian Protocol': a simplified head-up tilt testing potentiated with oral nitroglycerin to assess patients with unexplained syncope. Europace.

[19] Eeftinck Schattenkerk DW, van Lieshout JJ, van den Meiracker AH, Wesseling KR, Blanc S, Wieling W, van Montfrans GA, Settels JJ, Wesseling KH, Westerhof BE (2009). Nexfin noninvasive continuous blood pressure validated against Riva-Rocci/Korotkoff. Am J Hypertens.

[20] Sheldon RS, Grubb BP, Olshansky B, Shen WK, Calkins H, Brignole M, Raj SR, Krahn AD, Morillo CA, Stewart JM (2015). Heart rhythm society expert consensus statement on the diagnosis and treatment of postural tachycardia syndrome, inappropriate sinus tachycardia, and vasovagal syncope. Heart Rhythm 2015.

[21] Ricci F, De Caterina R, Fedorowski A (2015). Orthostatic hypotension: epidemiology, prognosis, and treatment. J Am Coll Cardiol.

[22] Spahic JM, Ricci F, Aung N, Axelsson J, Melander O, Sutton R, Hamrefors V, Fedorowski A. Proconvertase furin is downregulated in postural orthostatic tachycardia syndrome. Front Neurosci. 2019;13(301). 10.3389/fnins.2019.00301. 10.3389/fnins.2019.00301 PMC6455076 31001074

[23] Marshall A, Altman DG, Holder RL, Royston P (2009). Combining estimates of interest in prognostic modelling studies after multiple imputation: current practice and guidelines. BMC Med Res Methodol.

[24] Gladitz J, Rubin DB (1989). Multiple Imputation for Nonresponse in Surveys. John Wiley & Sons, Chichester – New York – Brisbane – Toronto – Singapore 1987, xxx, 258 S., 6 Abb., £ 30.25, ISSN 0271-6232. Biom J.

[25] Muller EE, Locatelli V, Cocchi D (1999). Neuroendocrine control of growth hormone secretion. Physiol Rev.

[26] Hallengren E, Almgren P, Engstrom G, Hedblad B, Persson M, Suhr J, Bergmann A, Melander O (2014). Fasting levels of high-sensitivity growth hormone predict cardiovascular morbidity and mortality: the Malmo diet and Cancer study. J Am Coll Cardiol.

[27] O'Connor JC, McCusker RH, Strle K, Johnson RW, Dantzer R, Kelley KW (2008). Regulation of IGF-I function by proinflammatory cytokines: at the interface of immunology and endocrinology. Cell Immunol.

[28] Okamoto LE, Raj SR, Gamboa A, Shibao CA, Arnold AC, Garland EM, Black BK, Farley G, Diedrich A, Biaggioni I (2015). Sympathetic activation is associated with increased IL-6, but not CRP in the absence of obesity: lessons from postural tachycardia syndrome and obesity. Am J Physiol Heart Circ Physiol.

[29] Stewart JM, Taneja I, Medow MS (2007). Reduced body mass index is associated with increased angiotensin II in young women with postural tachycardia syndrome. Clin Sci (Lond).

[30] Pokrajac A, Frystyk J, Flyvbjerg A, Trainer PJ (2009). Pituitary-independent effect of octreotide on IGF1 generation. Eur J Endocrinol.

[31] Miller AJ, Raj SR (2018). Pharmacotherapy for postural tachycardia syndrome. Auton Neurosci.

[32] Stewart JM, Medow MS, Glover JL, Montgomery LD (2006). Persistent splanchnic hyperemia during upright tilt in postural tachycardia syndrome. Am J Physiol Heart Circ Physiol.

[33] Conti E, Carrozza C, Capoluongo E, Volpe M, Crea F, Zuppi C, Andreotti F (2004). Insulin-like growth factor-1 as a vascular protective factor. Circulation.

[34] Oldham WM, Lewis GD, Opotowsky AR, Waxman AB, Systrom DM (2016). Unexplained exertional dyspnea caused by low ventricular filling pressures: results from clinical invasive cardiopulmonary exercise testing. Pulm Circ.

[35] Lien K, Johansen B, Veierod MB, Haslestad AS, Bohn SK, Melsom MN, Kardel KR, Iversen PO (2019). Abnormal blood lactate accumulation during repeated exercise testing in myalgic encephalomyelitis/chronic fatigue syndrome. Physiol Rep.

[36] Albertsson-Wikland K, Rosberg S, Karlberg J, Groth T (1994). Analysis of 24-hour growth hormone profiles in healthy boys and girls of normal stature: relation to puberty. J Clin Endocrinol Metab.

[37] Engstrom BE, Karlsson FA, Wide L (1998). Marked gender differences in ambulatory morning growth hormone values in young adults. Clin Chem.

[38] Wauthier V, Sugathan A, Meyer RD, Dombkowski AA, Waxman DJ (2010). Intrinsic sex differences in the early growth hormone responsiveness of sex-specific genes in mouse liver. Mol Endocrinol.

[39] Veldhuis JD (1996). Gender differences in secretory activity of the human somatotropic (growth hormone) axis. Eur J Endocrinol.

[40] Ocon AJ, Medow MS, Taneja I, Clarke D, Stewart JM (2009). Decreased upright cerebral blood flow and cerebral autoregulation in normocapnic postural tachycardia syndrome. Am J Physiol Heart Circ Physiol.

[41] Joyner MJ, Masuki S (2008). POTS versus deconditioning: the same or different?. Clin Auton Res.

[42] Garland EM, Celedonio JE, Raj SR (2015). Postural tachycardia syndrome: beyond orthostatic intolerance. Curr Neurol Neurosci Rep.

[43] Jarjour IT, Jarjour LK (2013). Low iron storage and mild anemia in postural tachycardia syndrome in adolescents. Clin Auton Res.

[44] de Groot B, Zuurbier CJ, van Beek JH (1999). Dynamics of tissue oxygenation in isolated rabbit heart as measured with near-infrared spectroscopy. Am J Phys.

[45] Yu X, Li H, Murphy TA, Nuss Z, Liles J, Liles C, Aston CE, Raj SR, Fedorowski A, Kem DC. Angiotensin II Type 1 Receptor Autoantibodies in Postural Tachycardia Syndrome. J Am Heart Assoc. 2018;7(8). 10.1161/JAHA.117.008351. 10.1161/JAHA.117.008351 PMC6015435 29618472

[46] Tanaka M, Sato M, Umehara S, Nishikawa T (2003). Influence of menstrual cycle on baroreflex control of heart rate: comparison with male volunteers. Am J Physiol Regul Integr Comp Physiol.

