# Supplementary material for: Proteomic analysis reveals sex-specific biomarker signature in postural orthostatic tachycardia syndrome
Source: BMC Cardiovasc Disord. 2020 Apr 22;20:190. doi: 10.1186/s12872-020-01465-6 (PMC7178975; doi:10.1186/s12872-020-01465-6)
Supplement: Supplementary file 2 — Additional file 2: Table S1. Absolute and percentage missingness data. Table S2. Multiplex Cardiovascular Disease Panel: Biomarker List [file 12872_2020_1465_MOESM2_ESM.docx]

**Table S1. Absolute and percentage missingness data.**

| **Characteristic** | **POTS -**  **missing** | **POTS -**  **missing percent** | **POTS +**  **missing** | **POTS +**  **missing percent** |
| --- | --- | --- | --- | --- |
| Age | 0 | 0 | 0 | 0 |
| Sex | 0 | 0 | 0 | 0 |
| BMI | 0 | 0 | 0 | 0 |
| Systolic BP supine | 2 | 0,71 | 0 | 0 |
| Diastolic BP supine | 2 | 0,71 | 0 | 0 |
| Heart rate supine | 2 | 0,71 | 0 | 0 |
| Systolic BP HUT min | 2 | 0,71 | 0 | 0 |
| Diastolic BP HUT min | 2 | 0,71 | 0 | 0 |
| Hearte rate HUT min | 2 | 0,71 | 0 | 0 |
| Smoking | 0 | 0 | 0 | 0 |
| IL8 | 94 | 33,22 | 36 | 31,86 |
| VEGFA | 94 | 33,22 | 36 | 31,86 |
| AM | 95 | 33,57 | 36 | 31,86 |
| CD40L | 94 | 33,22 | 36 | 31,86 |
| GDF15 | 94 | 33,22 | 36 | 31,86 |
| SELE | 94 | 33,22 | 36 | 31,86 |
| EGF | 94 | 33,22 | 36 | 31,86 |
| OPG | 94 | 33,22 | 36 | 31,86 |
| SRC | 94 | 33,22 | 36 | 31,86 |
| IL1ra | 94 | 33,22 | 36 | 31,86 |
| IL6 | 94 | 33,22 | 36 | 31,86 |
| CSTB | 94 | 33,22 | 36 | 31,86 |
| KLK6 | 94 | 33,22 | 36 | 31,86 |
| Gal3 | 94 | 33,22 | 36 | 31,86 |
| PAR1 | 94 | 33,22 | 36 | 31,86 |
| TRAIL | 94 | 33,22 | 36 | 31,86 |
| TIE2 | 94 | 33,22 | 36 | 31,86 |
| TF | 94 | 33,22 | 36 | 31,86 |
| TNFR1 | 94 | 33,22 | 36 | 31,86 |
| PDGFsubunitB | 94 | 33,22 | 36 | 31,86 |
| CSF1 | 94 | 33,22 | 36 | 31,86 |
| CXCL1 | 94 | 33,22 | 36 | 31,86 |
| LOX1 | 94 | 33,22 | 36 | 31,86 |
| TRAILR2 | 94 | 33,22 | 36 | 31,86 |
| FGF23 | 94 | 33,22 | 36 | 31,86 |
| SCF | 94 | 33,22 | 36 | 31,86 |
| IL18 | 94 | 33,22 | 36 | 31,86 |
| IL6RA | 94 | 33,22 | 36 | 31,86 |
| TNFR2 | 94 | 33,22 | 36 | 31,86 |
| HSP27 | 95 | 33,57 | 38 | 33,63 |
| TNFSF14 | 94 | 33,22 | 36 | 31,86 |
| PRL | 94 | 33,22 | 36 | 31,86 |
| MPO | 94 | 33,22 | 36 | 31,86 |
| GH | 94 | 33,22 | 36 | 31,86 |
| MMP1 | 94 | 33,22 | 36 | 31,86 |
| RETN | 94 | 33,22 | 36 | 31,86 |
| FAS | 94 | 33,22 | 36 | 31,86 |
| PAPPA | 101 | 35,69 | 36 | 31,86 |
| PTX3 | 95 | 33,57 | 36 | 31,86 |
| REN | 94 | 33,22 | 36 | 31,86 |
| CHI3L1 | 94 | 33,22 | 36 | 31,86 |
| ST2 | 94 | 33,22 | 36 | 31,86 |
| TRANCE | 94 | 33,22 | 37 | 32,74 |
| HGF | 94 | 33,22 | 36 | 31,86 |
| PSGL1 | 97 | 34,28 | 37 | 32,74 |
| MB | 94 | 33,22 | 36 | 31,86 |
| TM | 94 | 33,22 | 36 | 31,86 |
| IL16 | 94 | 33,22 | 36 | 31,86 |
| MMP10 | 94 | 33,22 | 36 | 31,86 |
| UPAR | 94 | 33,22 | 36 | 31,86 |
| CCL4 | 94 | 33,22 | 36 | 31,86 |
| CTSD | 94 | 33,22 | 36 | 31,86 |
| RAGE | 94 | 33,22 | 36 | 31,86 |
| CCL3 | 95 | 33,57 | 36 | 31,86 |
| MMP7 | 94 | 33,22 | 36 | 31,86 |
| CXCL6 | 94 | 33,22 | 36 | 31,86 |
| ITGB1BP2 | 97 | 34,28 | 38 | 33,63 |
| CXCL16 | 94 | 33,22 | 36 | 31,86 |
| Dkk1 | 94 | 33,22 | 36 | 31,86 |
| SIRT2 | 94 | 33,22 | 38 | 33,63 |
| GAL | 94 | 33,22 | 36 | 31,86 |
| AGRP | 94 | 33,22 | 36 | 31,86 |
| CD40 | 94 | 33,22 | 36 | 31,86 |
| tPA | 94 | 33,22 | 36 | 31,86 |
| HBEGF | 94 | 33,22 | 36 | 31,86 |
| ESM1 | 94 | 33,22 | 36 | 31,86 |
| VEGFD | 94 | 33,22 | 36 | 31,86 |
| MMP12 | 94 | 33,22 | 36 | 31,86 |
| SPON1 | 94 | 33,22 | 36 | 31,86 |
| CASP8 | 95 | 33,57 | 39 | 34,51 |
| CTSL1 | 94 | 33,22 | 36 | 31,86 |
| CX3CL1 | 94 | 33,22 | 36 | 31,86 |
| LEP | 99 | 34,98 | 40 | 35,4 |
| CCL20 | 94 | 33,22 | 36 | 31,86 |
| CA125 | 94 | 33,22 | 36 | 31,86 |
| NEMO | 94 | 33,22 | 36 | 31,86 |
| FS | 94 | 33,22 | 36 | 31,86 |
| PECAM1 | 94 | 33,22 | 36 | 31,86 |
| ECP | 95 | 33,57 | 36 | 31,86 |
| IL27A | 94 | 33,22 | 36 | 31,86 |
| hK11 | 94 | 33,22 | 36 | 31,86 |
| MCP1 | 94 | 33,22 | 36 | 31,86 |
| PlGF | 94 | 33,22 | 36 | 31,86 |
| TIM | 94 | 33,22 | 36 | 31,86 |

**Table S2. Multiplex Cardiovascular Disease Panel: Biomarker List**

Adrenomedullin (AM)

Agouti-related protein (AGRP)

Angiopoietin-1 receptor (TIE2)

Beta-nerve growth factor (Beta-NGF)

Caspase-8 (CASP-8)

Cathepsin D (CTSD)

Cathepsin L1 (CTSL1)

C-C motif chemokine 3 (CCL3

C-C motif chemokine 4 (CCL4)

C-C motif chemokine 20 (CCL20)

CD40 ligand (CD40L)

Chitinase-3-like protein 1 (CHI3L1)

C-X-C motif chemokine 1 (CXCL1)

C-X-C motif chemokine 6 (CXCL6)

C-X-C motif chemokine 16 (CXCL16)

Cystatin-B (CSTB)

Dickkopf-related protein 1 (Dkk-1)

Endothelial cell-specific molecule 1 (ESM-1)

Eosinophil cationic protein (ECP)

Epidermal growth factor (EGF)

E-selectin (SELE)

Fatty acid-binding protein, adipocyte (FABP4)

Fibroblast growth factor 23 (FGF-23) Follistatin (FS)

Fractalkine (CX3CL1) Galanin peptides (GAL) Galectin-3 (Gal-3)

Growth hormone (GH)

Growth/differentiation factor 15 (GDF-15)

Extension Control (Ext Ctrl)

Heat shock 27 kDa protein (HSP 27)

Heparin-binding EGF-like growth factor (HB-EGF)

Hepatocyte growth factor (HGF)

Interleukin-1 receptor antagonist protein (IL-1ra)

Interleukin-4 (IL-4)

Interleukin-6 (IL-6)

Interleukin-6 receptor subunit alpha (IL-6RA)

Interleukin-8 (IL-8)

Incubation Control (Inc Ctrl 1)

Interleukin-16 (IL-16)

Interleukin-18 (IL-18)

Interleukin-27 (IL-27)

Kallikrein-6 (KLK6)

Kallikrein-11 (hk11)

Lectin-like oxidized LDL receptor 1 (LOX-1)

Leptin (LEP)

Macrophage colony-stimulating factor 1 (CSF-1)

Matrix metalloproteinase-1 (MMP-1)

Matrix metalloproteinase-3 (MMP-3)

Matrix metalloproteinase-7 (MMP-7)

Matrix metalloproteinase-10 (MMP-10)

Matrix metalloproteinase-12 (MMP-12)

Melusin (ITGB1BP2)

Membrane-bound aminopeptidase P (mAmP)

Monocyte chemotactic protein 1 (MCP)

Myeloperoxidase (MPO)

Myoglobin (MB)

Natriuretic peptides B (BNP)

NF-kappa-B essential modulator (NEMO)

N-terminal pro-B-type natriuretic peptide (NT-pro- BNP)

Osteoprotegerin (OPG)

Ovarian cancer-related tumor marker 125 (CA-125)

Pappalysin-1 (PAPPA)

Pentraxin-related protein PTX3 (PTX3)

Placenta growth factor (PlGF)

Platelet endothelial celll adhesion molecule (PECAM-1)

Detection Control (Det Ctrl)

Platelet-derived growth factor subunit B (PDGF subunit B)

Incubation Control (Inc Ctrl 2)

Prolactin (PRL)

Protein S100-A12 (EN-RAGE)

Proteinase-activated receptor 1 (PAR-1)

Proto-oncogene tyrosine-protein kinase Src (SRC)

P-selectin glycoprotein ligand 1 (PSGL-1)

Receptor for advanced glycosylation end products (RAGE)

Renin (REN)

Resistin (RETN)

SIR2-like protein (SIRT2)

Spondin-1 (SPON1)

ST2 protein (ST2)

Stem cell factor (SCF)

Thrombomodulin (TM)

TIM-1 (TIM)

Tissue factor (TF)

Tissue-type plasminogen activator (t-PA)

TNF-related activation-induced cytokine (TRANCE)

TNF-related apoptosis-inducing ligand (TRAIL)

Tumor necrosis factor ligand superfamily member 14 (TNFSF14)

Tumor necrosis factor receptor 1 (TNF-R1)

Tumor necrosis factor receptor 2 (TNF-R2)

TNF-related apoptosis-inducing ligand receptor 2 (TRAIL-R2)

Tumor necrosis factor receptor superfamily member 5 (CD40)

Tumor necrosis factor receptor superfamily member 6 (FAS)

Urokinase plasminogen activator surface receptor (U-PAR)

Vascular endothelial growth factor A (VEGF-A)

Vascular endothelial growth factor D (VEGF-D)
